# VIEWS ON HELPFUL SUBSTANCE USE RECOVERY PATHWAYS: DO OLDER AMERICANS DIFFER FROM THEIR YOUNGER COUNTERPARTS?

**DOI:** 10.1093/geroni/igad104.2521

**Published:** 2023-12-21

**Authors:** Naomi Meier, Rachita Sharma, Paula Garland, Ami Moore

**Affiliations:** University of North Texas, Denton, Texas, United States; University of North Texas, Denton, Texas, United States; University of North Texas, Denton, Texas, United States; University of North Texas: Denton, Denton, Texas, United States

## Abstract

Recovery from substance use is generally a lifelong process with different pathways. This study used data from the Justice Community Opioid Innovation Network (JCOIN), 2021, a nationally representative survey of over 6,000 American adults, to examine factors that influence the views on most helpful pathways for recovery among older Americans (60 years and older) compared to their younger counterparts (less than 60 years old). Multiple linear regression was used to determine differences across age, political affiliation, cultural identity, and regional location. A factor analysis of 9 questions that asked respondents to rate the most helpful pathways for recovery generated three domains: 1. Medical services enabling environment (PCP, intensive inpatient program, and rehab program); 2. Direct conventional treatment (Prescription medication and therapist); and 3. Sociocultural factors/non-conventional treatment (Family/friends, spiritual/natural healer, self-help group, and faith-based organization). Older and younger Democrats, Independents, and residents of the Northeast and Midwest regions agreed that the medical services were helpful for recovery. While younger and older Democrats believed that direct conventional treatment was helpful, younger and older Republics as well as younger married people believed it to be less helpful. While older Democrats agreed that non-conventional treatments are most helpful, younger Democrats and Independents believed them to be less helpful. However, younger Republicans, Blacks and Hispanics believed that they are most helpful. Given the significant differences in the views of older and younger Americans on helpful pathways to recovery, policymakers and stakeholders are encouraged to be mindful of the several policy recommendations presented in the paper.

